# The extracellular protease AdamTS-B is a dosage-sensitive regulator of unicellular tracheal branch morphogenesis

**DOI:** 10.1242/bio.062602

**Published:** 2026-08-14

**Authors:** Abigail Thuringer, Elizabeth Steinmetz, Muna Abdullahi, Afshan Ismat

**Affiliations:** Department of Biology, University of St. Thomas, Saint Paul, MN 55105, USA

**Keywords:** AdamTS-B, ECM remodeling, Tracheal morphogenesis

## Abstract

Epithelial tube morphogenesis requires coordinated interactions between epithelial cells and their extracellular environment, yet the extracellular mechanisms that regulate this process remain poorly understood. The *Drosophila* trachea provides an excellent model for investigating how extracellular remodeling contributes to epithelial tube morphogenesis. AdamTS-B, an extracellular ADAMTS protease, is expressed in the embryonic trachea from early to late stages of tracheal development. Loss of *AdamTS-B* caused mild defects in unicellular branch organization, whereas overexpression produced ectopic cyst-like structures. Additionally, AdamTS-B localized intracellularly and to basal protrusions of tracheal cells. Constitutively active EGFR phenocopied the *AdamTS-B* overexpression phenotype. Together, our findings identify AdamTS-B as a dosage-sensitive regulator of unicellular tracheal branch morphogenesis and support a model in which extracellular proteolysis contributes to epithelial tube architecture. The phenotypic similarity between *AdamTS-B* overexpression and constitutive EGFR activation further suggests a potential functional relationship between extracellular proteolysis and EGFR signaling during tracheal development.

## Teaser

**Summary:** Precise regulation of the extracellular protease AdamTS-B is required for proper epithelial tube morphogenesis and suggests a functional relationship with EGFR signaling.

## INTRODUCTION

During embryogenesis, epithelial cells undergo coordinated rearrangements to generate functional epithelial tubes. Epithelial tube morphogenesis requires coordinated communication between intracellular signaling pathways and the extracellular environment. Although it is well known that the extracellular matrix (ECM) provides signals to cells and tissues, as well as getting restructured for proper development, there is still much we do not know about the mechanisms by which the ECM and its remodeling influence epithelial tube formation (Rozario and DeSimone, 2010).

**Fig. 1.**
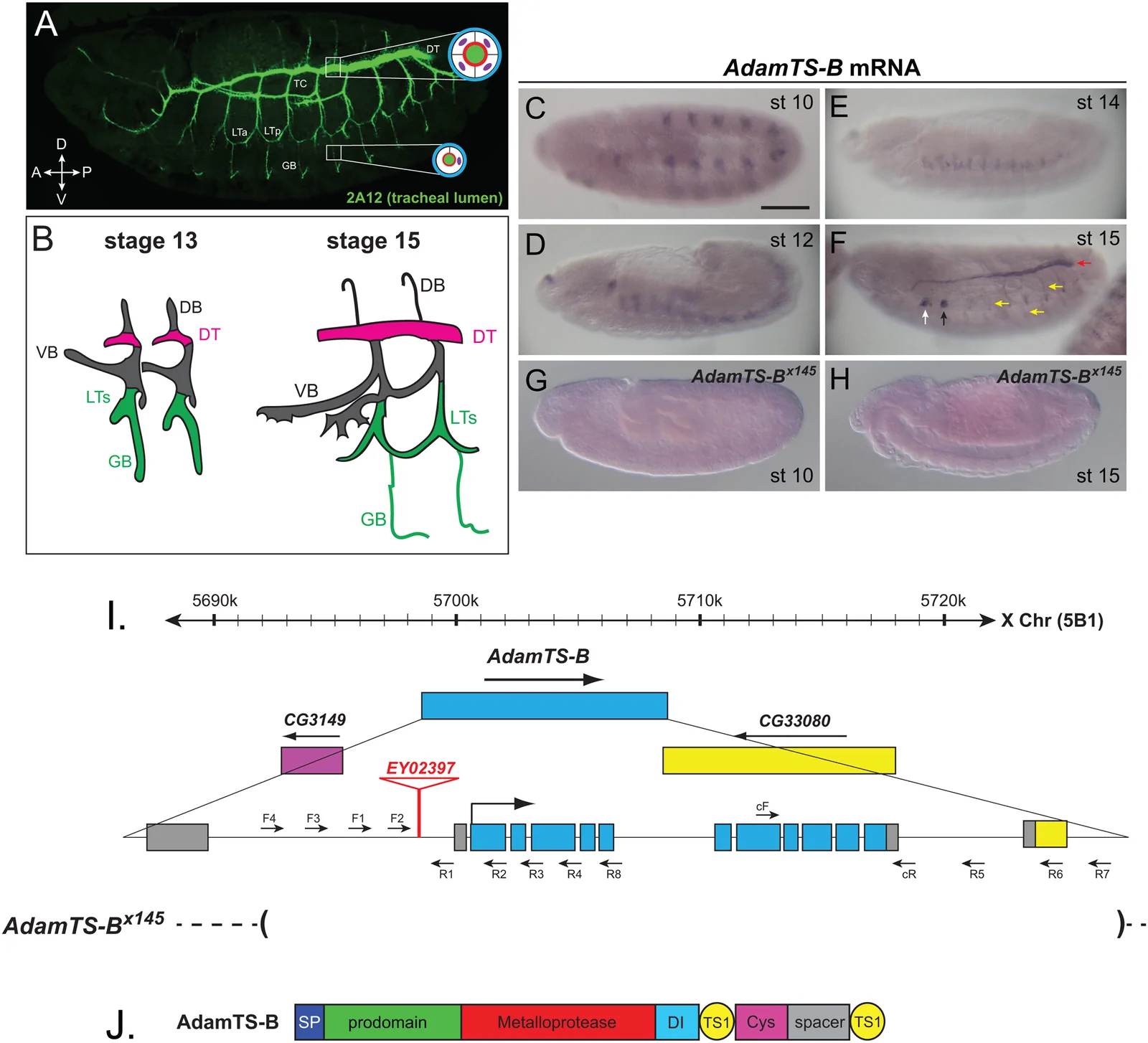
***AdamTS-B* is an extracellular protease expressed in the embryonic trachea.** (A) Stage 15 WT embryo stained with 2A12, a tracheal luminal marker to show the lumen of all tracheal tubes. The following branches/connections are labeled: dorsal trunk (DT), transverse connective (TC), anterior lateral trunk (LTa), posterior lateral trunk (LTp), and ganglionic branch (GB). The DT is a multicellular tube (top cross-sectioned tube) made of several epithelial cells with a lumen (green), a basal ECM (blue) and apical ECM (red). The GB is an example of a unicellular tube (bottom cross-sectioned tube) made of one cell wrapped around itself, also contains a lumen (green), a basal ECM (blue) and apical ECM (red). (B) Cartoon schematics of two tracheal metameres at stage 13 (during branch migration) and 15 (after migration, cell intercalation and elongation of branches). The multicellular DT is dark pink and the unicellular LTs and GBs are green. The visceral branches (VB) are also shown. The orientation of all embryos is shown as dorsal (D) top, ventral (V) bottom, anterior (A) left, and posterior (P) right. (C-H) *AdamTS-B* mRNA is expressed in the developing trachea starting at stage 10 in the tracheal placodes (C), stage 12 as tracheal cells start to migrate (D), stage 14 (E), and stage 15 in the main dorsal trunk (DT) (red arrow) and unicellular branches (yellow arrows). *AdamTS-B* mRNA is also expressed in the haltere (white arrow) and wing (black arrow) imaginal disc precursor cells (F). *AdamTS-B* mRNA is completely absent in *AdamTS-B^x145^* mutants at stage 10 (G) and stage 15 (H). (I) *AdamTS-B* is located at the 5B1 region of the X chromosome, between *CG3149* and *CG33080*. The black arrow below each gene name signifies the direction of transcription. The open reading frame (ORF) of *AdamTS-B* is composed of eleven exons (blue boxes). The P-element *EP02397* was used for an imprecise excision of the *AdamTS-B* ORF. Primers used for PCR of the excision are shown as black arrows. Based on PCR analysis, the extent of the possible deletion in *AdamTS-B^x145^* mutants is shown between the parentheses. (J) The predicted protein domain structure of AdamTS-B includes a Signal Peptide (SP), a prodomain, a Metalloprotease domain, a Disintegrin domain (DI), a Thrombospondin-like I repeat (TS1), a Cysteine-rich domain (Cys), an ADAM spacer domain (spacer), and a second TS1 domain. Scale bar: 100 µm.

The *Drosophila* trachea is a genetically tractable model for studying epithelial tube morphogenesis because of its highly stereotyped architecture and well-defined developmental program (Hayashi and Kondo, 2018; Maruyama and Andrew, 2012; Ochoa-Espinosa and Affolter, 2012) (Fig. 1A). The trachea forms through processes of invagination and migration followed by lumen formation and tube elongation (Andrew and Ewald, 2010). Each abdominal segment shares an identical tracheal branch pattern (except for the visceral branches) (Fig. 1A,B).

Epithelial tubes like the trachea are surrounded by a basement membrane, a specialized ECM on their basal sides (blue line, Fig. 1A). The basal ECM is deposited at the stage when branches start to grow in both diameter and length, after initial patterning and branching. The basal ECM regulates tracheal tube length by controlling cell shape changes associated with tube elongation (Klußmann-Fricke et al., 2022). These findings suggest that remodeling of the basal ECM contributes to the cell shape changes required for tube elongation. These tubes also contain an ECM on the apical side lining the lumen of the tubes, called the apical ECM (aECM) (Dong and Hayashi, 2015; Jaźwińska et al., 2003; Tonning et al., 2005; Luschnig et al., 2006; Luschnig and Uv, 2014; Öztürk-Çolak et al., 2016) (red line, Fig. 1A). Tracheal tubes are also lined by an aECM, composed primarily of chitin and associated proteins such as Piopio and Dumpy, which regulate lumen maturation and tube size (Wilkin et al., 2000; Jaźwińska et al., 2003; Tonning et al., 2005; Luschnig et al., 2006; Wang et al., 2006; Loganathan et al., 2020). However, the mechanisms that remodel the basal ECM during tracheal development and the contribution of this remodeling to epithelial tube morphogenesis remain poorly understood.

The trachea is composed of three different types of tubes: (1) multicellular tubes, (2) unicellular tubes, and (3) seamless tubes (Ghabrial et al., 2003; Hayashi and Kondo, 2018; Schottenfeld et al., 2010). Multicellular tubes form from multiple cells connected to each other on their lateral sides through adherens junctions. The lumen faces the apical surface of the cells. The main multicellular tube is the dorsal trunk (DT) that runs the anterior-posterior length of the embryo (Fig. 1A). Unicellular tubes form from one cell wrapping around the lumen and connecting to itself through an adherens junction. The dorsal branches (DBs), lateral trunks (LTs), and ganglionic branches (GBs) are examples of unicellular tubes (Fig. 1A). The terminal branches are examples of seamless tubes (Hayashi and Kondo, 2018; Schottenfeld et al., 2010). The unicellular tubes elongate through a process called cell intercalation where two cells start out next to each other and one cell slides past the other, resulting in the two cells end to end (Dong and Hayashi, 2015; Ribeiro et al., 2004; Schottenfeld et al., 2010). Whether remodeling of the basal ECM contributes to elongation of unicellular branches remains unknown. Extracellular proteases are well positioned to remodel the basal ECM during morphogenesis, yet their roles in tracheal tube development remain poorly understood.

The ADAMTS family of extracellular proteases in *Drosophila* have been shown to play important roles in a variety of morphogenetic processes. We previously shown that AdamTS-A functions to detach the trailing edge of salivary gland cells from the aECM to allow them to migrate forward in a smooth manner (Ismat et al., 2013). We also demonstrated an essential role for AdamTS-B in wing vein formation in the adult wing (Pham et al., 2018). In the wing, AdamTS-B was shown to effect cell fate as a negative regulator of wing vein formation. Previous studies suggested that AdamTS-B may regulate EGFR signaling (Butchar et al., 2012). Therefore, could the trachea also utilize an ADAMTS protease to remodel the basal ECM to regulate proper tube formation?

Here, we identify AdamTS-B as a dosage-sensitive regulator of unicellular tracheal branch morphogenesis. We show that precise regulation of AdamTS-B activity is required for normal branch architecture and that AdamTS-B localizes to basal protrusions of tracheal cells. Finally, constitutive EGFR activation phenocopies AdamTS-B overexpression, suggesting a potential functional relationship between extracellular proteolysis and EGFR signaling during epithelial tube morphogenesis.

## RESULTS

### *AdamTS-B* is an extracellular protease expressed in the embryonic trachea

AdamTS-B is one of three ADAMTS family proteases encoded in the *Drosophila* genome (Ozdowski et al., 2009; Ismat et al., 2013; Lhamo and Ismat, 2015; Skeath et al., 2017; Pham et al., 2018; Hamilton et al., 2022; Töpfer et al., 2024). *AdamTS-B* mRNA was first detected in all tracheal placodes at stage 10 and remained expressed throughout embryonic tracheal development (Fig. 1C-F). In addition to the trachea, *AdamTS-B* was expressed in the wing and haltere imaginal disc precursor cells (Pham et al., 2018) (black and white arrows, respectively, Fig. 1F). *AdamTS-B* encodes a predicted secreted metalloprotease containing the conserved domains characteristic of ADAMTS family proteins (Fig. 1J). We previously showed that AdamTS-B regulates wing vein formation (Pham et al., 2018). Here we investigate its function during embryonic tracheal development.

We generated a loss-of-function allele of AdamTS-B by imprecise excision of the EY02397 P-element (Fig. 1I). PCR mapping indicated that the *AdamTS-B^x145^* deletion removes the entire *AdamTS-B* coding region and may extend into the neighboring gene *CG33080*. Because PCR mapping was performed using heterozygous animals, the precise deletion boundaries could not be determined (Table S1). To further confirm we had generated a deletion of *AdamTS-B*, we examined *AdamTS-B* expression by *in situ* hybridization. The resulting deletion mutation (*AdamTS-B^x145^*) was the only deletion that displayed a complete lack of *AdamTS-B* mRNA expression (Fig. 1G,H compared to wild-type expression at comparable stages in Fig. 1C,F). Among the deletion alleles examined, only *AdamTS-B^x145^* completely lacked *AdamTS-B* mRNA expression, indicating that it represents a strong loss-of-function allele (Fig. 1G,H). We next used the *AdamTS-B^x145^* allele to determine the role of AdamTS-B during embryonic tracheal morphogenesis.

### AdamTS-B contributes to proper tracheal branch morphogenesis

**Fig. 2.**
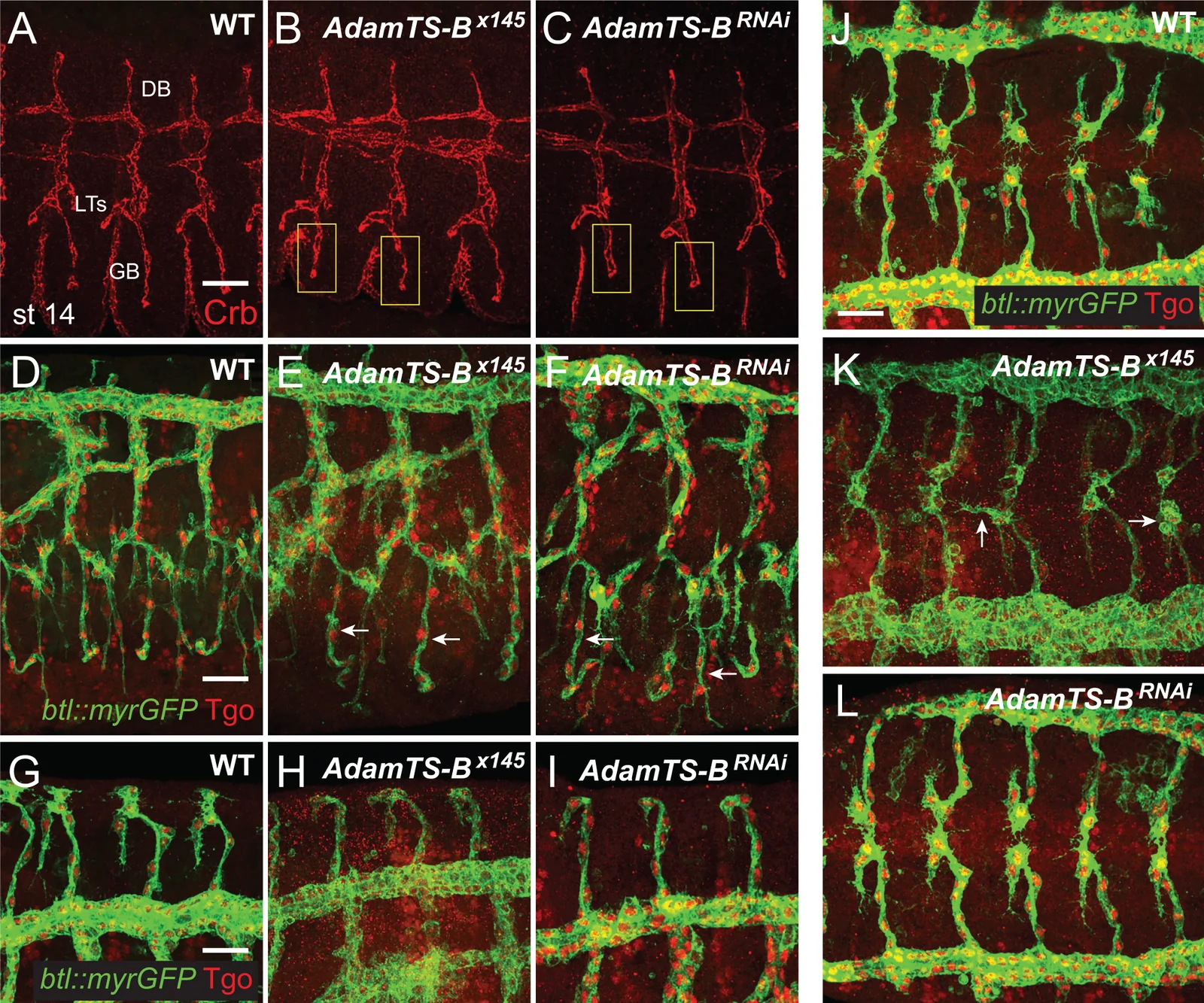
**AdamTS-B contributes to proper tracheal branch morphogenesis.** (A-C) Stage 14 WT, *AdamTS-B^x145^*, and *AdamTS-B^RNAi^* (*btl*-GAL4::*UAS-AdamTS-B^RNAi^*) embryos stained with anti-Crb (red) to mark the apical membranes of all tracheal cells. A. Lateral view of WT embryo showing tracheal branches in three hemisegments. dorsal branch (DB), lateral transverse branches (LTs), and ganglionic branches (GB) are labeled. (B) *AdamTS-B^x145^* embryo showing no difference in DBs, LTs, and slightly shorter GBs (yellow boxes). (C) *AdamTS-B^RNAi^* embryo showing no difference in DBs, LTs, and slightly shorter GBs (yellow boxes). (D-L) Stage 16 wild type (WT), *AdamTS-B^x145^*, and *AdamTS-B^RNAi^* embryos marked with a myristoylated GFP (myrGFP) in the trachea (*btl::myrGFP*) (green) and Tgo (red) to mark tracheal nuclei. (D) Lateral view of WT embryo showing tracheal branches in three hemisegments. dorsal trunk (DT), visceral branch (VBs), and GBs are labeled. (E) *AdamTS-B^x145^* embryo showing slightly disorganized branching (white arrows). The GBs appear similar in length compared to WT. (F) *AdamTS-B^RNAi^* embryo showing slightly disorganized branching (white arrows). The GBs appear similar in length compared to WT. (G) Lateral view of WT embryo showing DBs after completion of cell intercalation. (H) *AdamTS-B^x145^* embryo showing DBs that do not extend as far ventrally as in WT (white arrows). (I) *AdamTS-B^RNAi^* embryo showing DBs that do not extend as far ventrally as in WT (white arrows). Dorsal view of WT embryo showing DBs from both later sides meeting each other. (K) *AdamTS-B^x145^* embryo showing slightly misshapen DBs as they meet (white arrows). (L) *AdamTS-B^RNAi^* embryo showing DBs similar to WT. Scale bars: 20 µm.

To determine how AdamTS-B regulates tracheal tube formation, we stained wild-type (WT), *AdamTS-B^x145^* (null mutant), and tracheal-specific *AdamTS-B^RNAi^* embryos with the apical tracheal marker Crumbs (Crb) (Fig. 2). *AdamTS-B^RNAi^* was expressed specifically in the trachea using *btl*-GAL4 to determine whether any observed phenotypes were due to loss of *AdamTS-B* within tracheal cells. Crb marks the apical domain of tracheal cells and is an early tracheal marker (stage 9 to 15). Using Crb we examined the trachea of stage 14 embryos in WT, *AdamTS-B^x145^*, and *AdamTS-B^RNAi^* embryos. At stage 14, the ganglionic branch (GB) was slightly shorter in both *AdamTS-B^x145^*, and *AdamTS-B^RNAi^* compared to WT (yellow boxes in Fig. 2B,C compared to WT in Fig. 2A). Because the GBs appeared shorter at stage 14, we next asked whether this early phenotype persisted through later stages of tracheal development or reflected defects in branch elongation mediated by cell intercalation. Cell intercalation drives elongation of unicellular tracheal branches by rearranging neighboring cells from a side-by-side to an end-to-end configuration (Casani et al., 2020; Caussinus et al., 2008; Ribeiro et al., 2004). Using a myristoylated GFP (myrGFP) expressed in the trachea (*btl::myrGFP*) and a tracheal nuclear marker Tango (Tgo), we examined LTs and GBs at stage 16 when cell intercalation is complete. By stage 16, GB length and organization were indistinguishable from WT (Fig. 2E,F), suggesting that the earlier reduction in GB length is transient (white arrows in Fig. 2E,F). Because DBs provide the best-characterized model for tracheal cell intercalation, we next examined DB morphology in *AdamTS-B*-deficient embryos. No defects in cell intercalation were observed in the DBs of either *AdamTS-B^x145^* or *AdamTS-B^RNAi^* embryos (Fig. 2H,I). However, *AdamTS-B^x145^* embryos displayed mild disorganization of DB morphology (white arrows in Fig. 2K), indicating that overall branch architecture is affected despite apparently normal intercalation. Interestingly, tracheal-specific *AdamTS-B^RNAi^* produced more pronounced defects in the LTs and GBs, whereas *AdamTS-B^x145^* mutants exhibited milder LT/GB defects but greater DB disorganization. The basis for these differences is unclear but may reflect differences in the timing or efficiency of gene depletion or genetic background effects. Together, these observations indicate that AdamTS-B is not required for the major morphogenetic events of tracheal tube formation but contributes to proper branch organization during late embryonic development.

### *AdamTS-B* overexpression induces cyst-like structure formation in unicellular tracheal branches

**Fig. 3.**
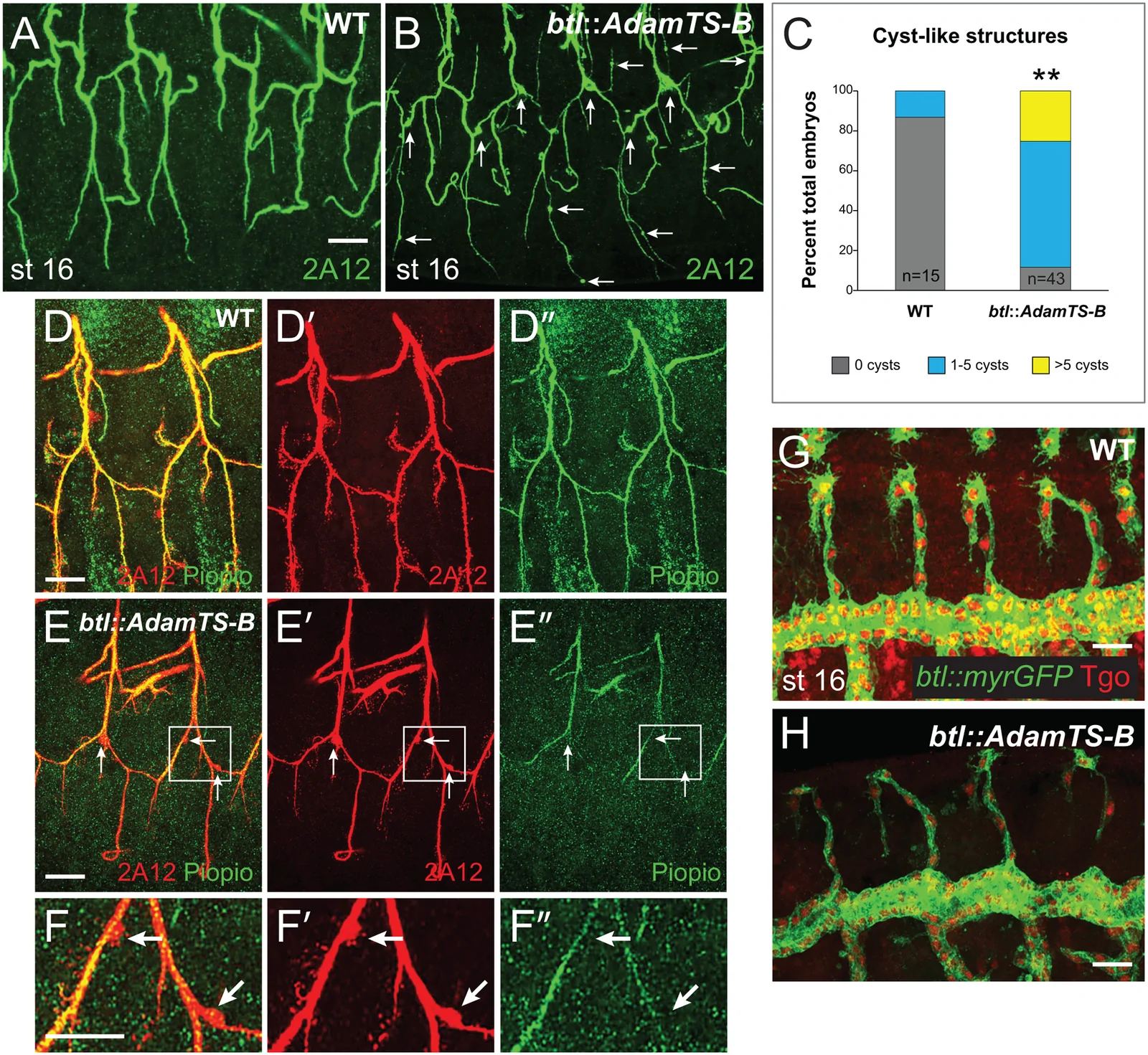
***AdamTS-B* overexpression induces cyst-like structure formation in unicellular tracheal branches.** *UAS-AdamTS-B* was expressed in the trachea using a tracheal-specific *btl*-GAL4 driver (*btl::AdamTS-B*) at 25°C. (A) Stage 16 WT embryo stained with 2A12 (green), a marker of the tracheal lumen, showing tracheal branches with uniform luminal diameter. (B) *AdamTS-B* overexpressed in the trachea (*btl::AdamTS-B*) displays branch cysts (white arrows). (C) Quantification of branch cysts in *AdamTS-B* overexpressed embryos (*btl::AdamTS-B*) compared to WT. **p<1×10^−6^ using a G-test of Independence. (D-D″) Stage 15 WT embryo stained with 2A12 (red) and Piopio (green), two luminal markers showing even distribution of Piopio in the tracheal lumen. (E-E″) *AdamTS-B* overexpressed embryo shows very little to no Piopio staining in the branch cysts (white arrows). (F-F″) Magnified views of boxed regions in E-E″ show more clearly no Piopio staining in the branch cysts (white arrows). (G) Lateral view of a stage 16 WT embryo marked with myrGFP in the trachea (*btl::myrGFP*) (green) and Tgo (red) to mark tracheal nuclei showing four DBs at the end of cell intercalation. (H) *AdamTS-B* overexpressed embryo showing normal DBs compared to WT. Scale bars: 20 µm.

**Fig. 4.**
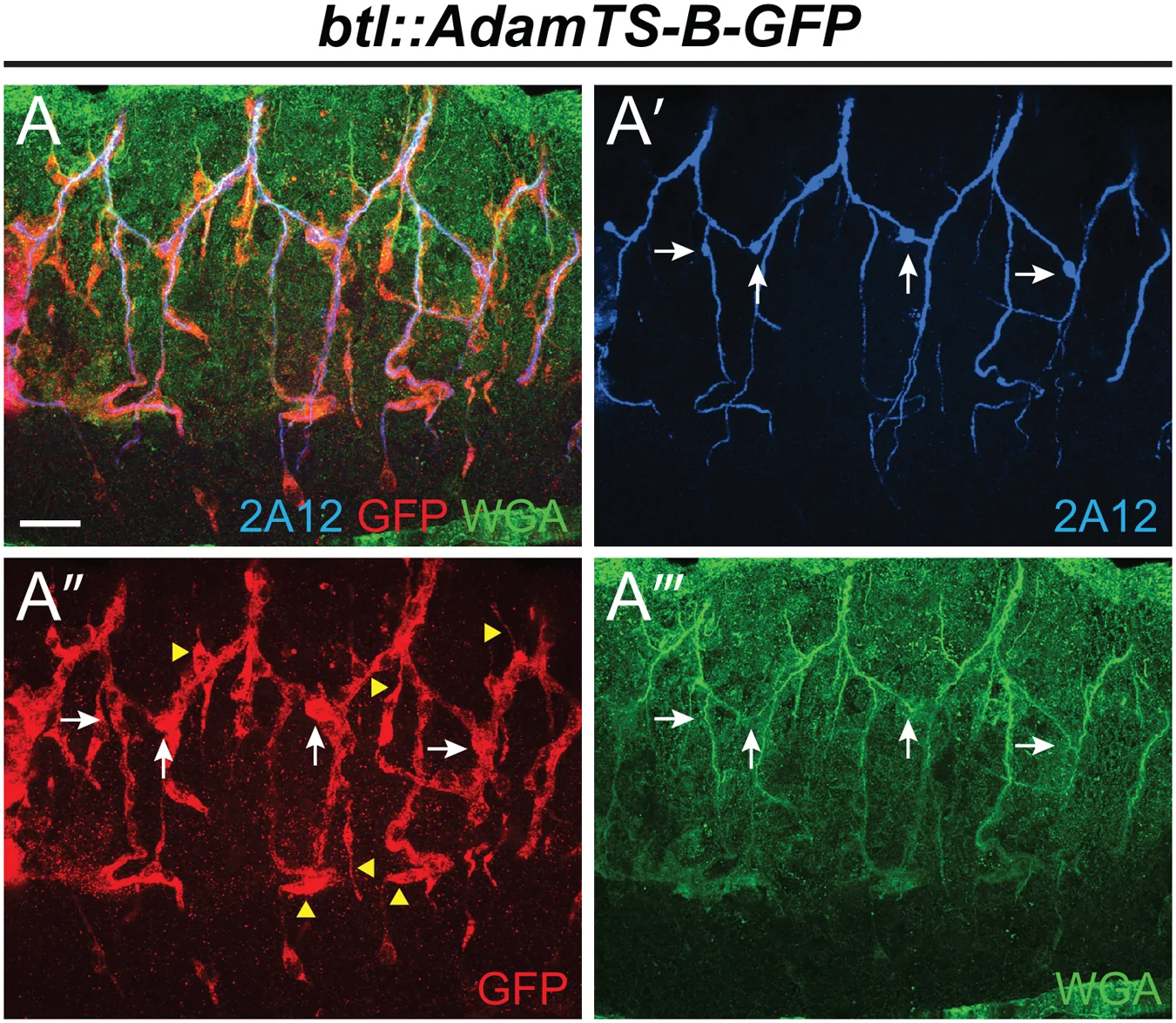
**GFP-tagged AdamTS-B accumulates intracellularly and within basal protrusions of tracheal cells.** (A) C-terminal GFP-tagged *UAS-AdamTS-B* was expressed in the trachea using a tracheal-specific *btl*-GAL4 driver (*btl::AdamTS-B-GFP*) at 25°C. (A-A‴) Stage 16 *btl::AdamTS-B-GFP* stained with GFP (red) to mark AdamTS-B-GFP, 2A12 (blue) to mark the tracheal lumen, and WGA-488 (green) to mark the tracheal lumen. AdamTS-B-GFP localizes intracellularly and in basal protrusions of tracheal branches (yellow arrowheads in A″). The branch cysts seen with 2A12 (white arrows in A′) do not express WGA (white arrows in A‴, but do seem to express increased AdamTS-B-GFP fluorescence (white arrows in A″). Scale bar: 20 µm.

Because loss of *AdamTS-B* produced only subtle defects in tracheal branch morphology, we next asked whether increasing AdamTS-B levels would perturb tracheal development. Interestingly, overexpressing *AdamTS-B* just in the trachea resulted in a more striking defect, cyst-like structures in unicellular branches. These cysts were first identified using the luminal marker 2A12 (white arrows in Fig. 3B). Quantification revealed a significant increase in branch cyst-like structures compared to WT. Most significantly, more than 20% of embryos overexpressing *AdamTS-B* displayed more than five cysts, compared to zero in WT (Fig. 4C). To determine whether these structures represented expansions of the tracheal lumen, we examined the luminal marker Piopio (Jaźwińska et al., 2003). Piopio was absent from the cysts, indicating that they are not luminal structures (white arrows in Fig. 3E-F″). These cyst-like structures were readily detected with the luminal marker 2A12. However, they lacked Piopio staining, indicating that they do not represent typical luminal dilations.

Because loss of *AdamTS-B* caused mild disorganization of dorsal branches, we next asked whether *AdamTS-B* overexpression similarly affected branch organization. Using *btl::myrGFP* and Tgo, the DBs in *AdamTS-B* overexpression are indistinguishable to WT (Fig. 3H compared to WT in Fig. 3G). One possibility is that excess AdamTS-B activity disrupts extracellular matrix remodeling, leading to localized instability or bulging of the apical membrane that gives rise to non-luminal cysts. Together with the loss-of-function analysis, these findings support a role for AdamTS-B in maintaining proper tracheal branch morphology. The formation of non-luminal cysts following *AdamTS-B* overexpression suggests that excess protease activity perturbs apical membrane organization or stability.

### GFP-tagged AdamTS-B accumulates intracellularly and within basal protrusions of tracheal cells

Because *AdamTS-B* loss and overexpression altered tracheal branch morphology, we next asked where AdamTS-B localizes within tracheal cells. As tracheal cells are surrounded by both an apical and basal extracellular matrix (Loganathan et al., 2020), determining the localization of AdamTS-B may provide insight into where it functions. Therefore, we expressed a C-terminal GFP-tagged *AdamTS-B* (*UAS-AdamTS-B-GFP*) just in the trachea using *btl*-GAL4 (*btl::AdamTS-B-GFP*). Using 2A12 as a marker of the tracheal lumen, AdamTS-B-GFP was detected within tracheal cells and was enriched in basal protrusions (yellow arrowheads in Fig. 4A″). Moreover, the cyst-like structures can be seen in these AdamTS-B-GFP overexpression embryos using 2A12 (white arrows in Fig. 4A′). Consistent with the absence of Piopio, the luminal marker WGA also failed to label the cyst-like structures (Tonning et al., 2005) (white arrows in Fig. 4A‴), suggesting further that these cyst-like structures are not merely luminal dilations, but some abnormal structure. AdamTS-B-GFP was enriched at the cyst-like structures (white arrows in Fig. 4A″). Together, these observations demonstrate that AdamTS-B accumulates intracellularly and within basal protrusions of tracheal cells and is enriched at the abnormal cyst-like structures induced by its overexpression. The enrichment of AdamTS-B in basal protrusions is consistent with a role in remodeling the basal extracellular environment during tracheal branch morphogenesis.

### *AdamTS-B* overexpression phenocopies constitutive EGFR activation in tracheal branches

**Fig. 5.**
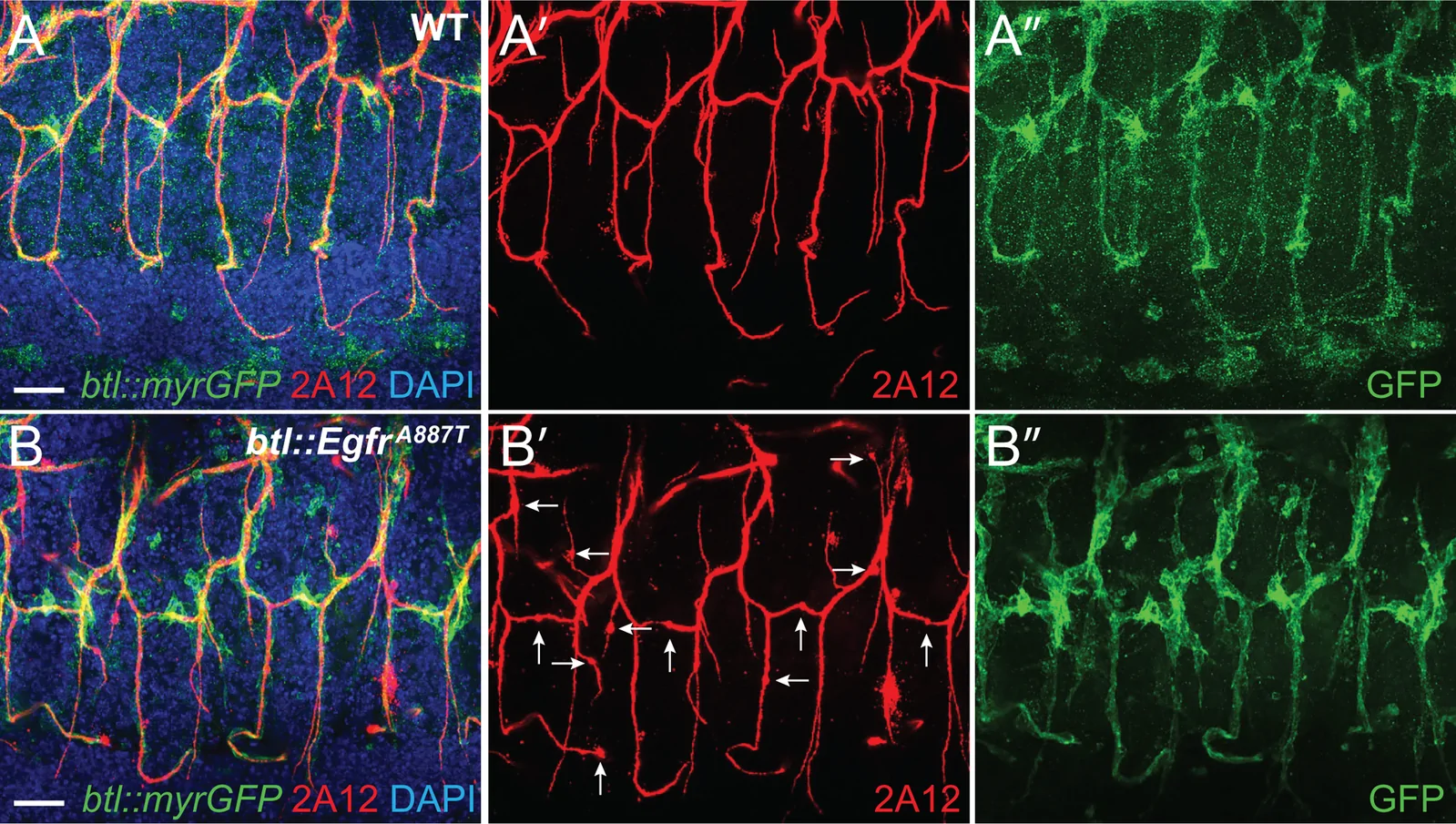
***AdamTS-B* overexpression phenocopies constitutive EGFR activation in tracheal branches.** (A) constitutively active version of Egfr (*UAS-Egfr^A887T^*) was expressed in the trachea using a tracheal-specific *btl*-GAL4 driver (*btl::Egfr^A887T^*) at 25°C. These embryos also expressed a myrGFP (*btl::myrGFP*) to mark tracheal cell membranes and were stained with 2A12 to mark the tracheal lumen and DAPI to mark all nuclei. (A-A″) Stage 16 WT embryo showing the lateral transverse (LT) branches with a uniform luminal diameter. (B-B″) Expressing the constitutively active version of the Egfr (*Egfr^A887T^*) in the trachea resulted in branch cysts throughout the trachea (white arrows in B′). Scale bars: 20 µm.

Previous studies implicated AdamTS-B in the regulation of EGFR signaling during wing vein formation (Butchar et al., 2012). We therefore asked whether increased EGFR activity produced tracheal defects similar to those caused by AdamTS-B overexpression. Tracheal-specific expression of constitutively active EGFR (*Egfr^A887T^*) resulted in cyst-like structures within unicellular branches that resembled those observed following *AdamTS-B* overexpression (Jeon and Zinn, 2009) (white arrows in Fig. 5B′). The phenotypic similarity between *AdamTS-B* overexpression and constitutive EGFR activation raises the possibility that AdamTS-B and EGFR signaling regulate a common process controlling membrane organization during unicellular branch morphogenesis. Collectively, our findings demonstrate that precise regulation of AdamTS-B activity is required for normal tracheal branch morphogenesis. The phenotypic similarity between *AdamTS-B* overexpression and constitutive EGFR activation further suggests that AdamTS-B may influence an EGFR-dependent process during tracheal development.

## DISCUSSION

**Fig. 6.**
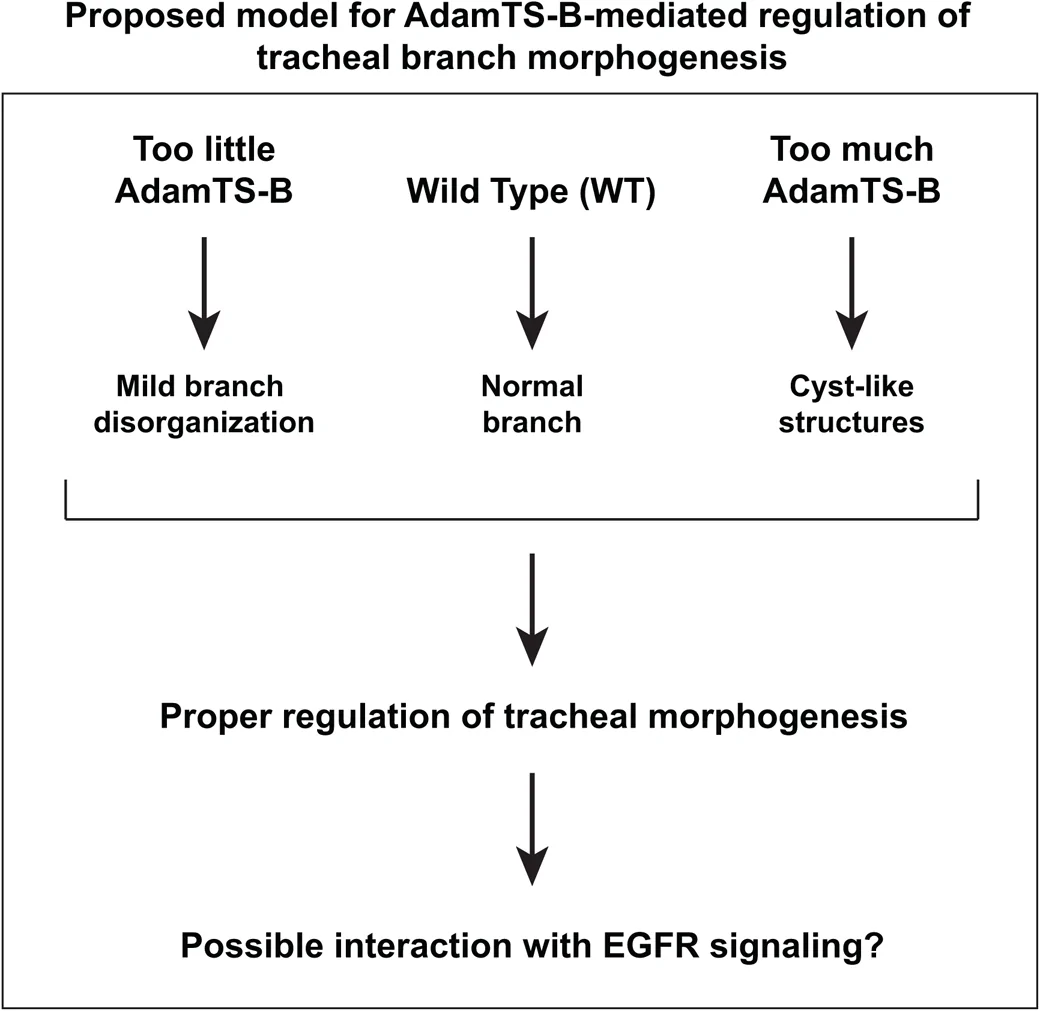
**Proposed model for AdamTS-B-mediated regulation of tracheal branch morphogenesis.** Under normal conditions, AdamTS-B localizes to basal protrusions, where it is proposed to remodel the basal extracellular environment. Loss of *AdamTS-B* results in mild branch disorganization, whereas excess AdamTS-B leads to the formation of cyst-like structures. The phenotypic similarity between *AdamTS-B* overexpression and constitutive EGFR activation suggests a possible interaction between AdamTS-B and EGFR signaling during tracheal morphogenesis, although the precise mechanism remains to be determined.

Our findings identify AdamTS-B as a novel regulator of unicellular tracheal branch morphogenesis. Together, the loss- and overexpression phenotypes support a model in which precise regulation of AdamTS-B activity is required for proper tracheal branch morphogenesis (Fig. 6). These observations further suggest that extracellular proteolysis contributes to epithelial tube morphogenesis and raise the possibility that AdamTS-B modulates EGFR-dependent signaling during tracheal development.

### AdamTS-B is a dosage-sensitive regulator of tracheal branch morphogenesis

Our overexpression data shows that excess AdamTS-B protease activity results in cyst-like structures in the trachea (Fig. 3). The marked difference between the loss- and overexpression phenotypes suggests that AdamTS-B activity must be precisely regulated during tracheal development. Such dosage sensitivity is consistent with extracellular proteases functioning as modulators of tissue architecture rather than binary developmental switches. We observed this same phenomenon in the posterior crossvein (PCV) of the wing where loss of *AdamTS-B* resulted in a significant increase, but not fully penetrant extra vein deltas in the PCV. Conversely, overexpression of *AdamTS-B* in the wing resulted in 100% PCVs completely absent (Pham et al., 2018).

### AdamTS-B likely functions by remodeling the extracellular environment surrounding unicellular branches

The enrichment of AdamTS-B-GFP within basal protrusions is consistent with AdamTS-B acting at the basal surface of tracheal cells. As we have not yet identified any substrate of AdamTS-B in the trachea, it is most likely that AdamTS-B cleaves an ECM protein that remodels the extracellular environment around the tracheal branches to allow for proper elongation and morphogenesis. Secondly, AdamTS-B could cleave cell-ECM connections that would allow the tracheal branches to form properly. A third possibility is that AdamTS-B functions directly with a known signaling pathway by either binding to and helping the receptor bind its ligands or it binds directly to one or more ligands to help them bind their receptor. More studies need to be done to parse out these possibilities.

### AdamTS-B may intersect with EGFR signaling to regulate membrane architecture and branch organization

The similarity between the phenotypes caused by AdamTS-B overexpression and constitutive EGFR activation suggests a possible functional relationship between AdamTS-B and EGFR signaling during tracheal morphogenesis. Specifically, in both AdamTS-B overexpression and constitutively active EGFR we observe cysts-like structures in unicellular branches (Figs 3 and 5). Because the cyst-like structures lacked Piopio and WGA, we think these abnormal structures are not merely luminal dilations (Figs 3 and 4). The similarity between the cyst-like structures produced by *AdamTS-B* overexpression and constitutive EGFR activation suggests that these two perturbations disrupt a common aspect of unicellular branch morphogenesis. However, AdamTS-B is a secreted protease, and we mostly observe this protease intracellularly and in basal protrusions (Fig. 4). One possibility is that AdamTS-B functions within the basal extracellular environment to modulate EGFR-dependent processes. Previous work implicated AdamTS-B in the regulation of EGFR signaling during wing vein formation, although AdamTS-B appeared to negatively regulate the pathway in that tissue (Butchar et al., 2012). Thus, AdamTS-B may modulate EGFR signaling in a tissue- or context-dependent manner. Alternatively, AdamTS-B and EGFR may act through parallel pathways that converge on a common process controlling unicellular branch architecture. Genetic interaction and pathway-activity experiments will be required to distinguish between these possibilities. It is interesting that previous studies on EGFR signaling in tracheal morphogenesis focused on either the DT (Olivares-Castiñeira and Llimargas, 2017) or the DBs (Cela and Llimargas, 2006). In both of these studies, as well as this study, EGFR signaling plays very different roles in tracheal morphogenesis. Together, these observations further support the idea that precise spatial and temporal regulation of EGFR signaling is required for multiple aspects of tracheal morphogenesis.

### Identifying the substrate(s) of AdamTS-B

Identifying the substrate(s) of AdamTS-B will be essential for understanding how this protease regulates tracheal morphogenesis. Candidate substrates include ECM proteins, cell adhesion molecules, or signaling components associated with the basal extracellular environment. Defining these targets will clarify whether AdamTS-B primarily remodels the extracellular matrix or instead modulates developmental signaling through proteolytic processing of signaling components.

### AdamTS-B function in wing vein formation versus tracheal morphogenesis

Our previous study on AdamTS-B focused on its role in the adult wing. Our surprising discovery was that, in the wing, AdamTS-B functions as a negative regulator in the cell fate decision of a wing cell to become a vein cell. This defect showed up mostly strikingly in the PCV (Pham et al., 2018). Cell fate decisions in the PCV are regulated by either the Egfr or Dpp signaling pathways (Bangi and Wharton, 2006; Blair, 2007; de Celis et al., 1996; Sturtevant et al., 1997). Butchar et al. (2012) hypothesized that AdamTS-B could possibly function through the Egfr pathway be sequestering one or more of the Egfr ligands like Spi, Vn, and Krn. Although AdamTS-B functions in cell fate decisions in the wing and cell intercalation in the trachea, we postulated that AdamTS-B may still function through the same signaling pathway in both tissues. As discussed above, our data in this study raise the possibility of an interaction between AdamTS-B and the Egfr pathway. Specifically, we show AdamTS-B to be functioning as a positive regulator of this pathway instead of a negative regulator in wing vein formation. This study identifies extracellular proteolysis as an important mechanism for regulating the architecture of unicellular epithelial tubes and suggests that ADAMTS proteases may influence morphogenesis by modulating conserved developmental signaling pathways.

## MATERIALS AND METHODS

### *Drosophila* stocks

The following *Drosophila melanogaster* stocks were used in this study: Canton S (CS) WT flies, *UAS-AdamTS-B* (Pham et al., 2018), *UAS-AdamTS-B-GFP* (Pham et al., 2018), *btl*-GAL4 (2nd chr.) (Andrew lab), *UAS-myr-GFP* (2nd chr.), *UAS-AdamTS-B^RNAi^* (3rd chr. – BL-44522)*, AdamTS-B^x145^* (see Generation of *AdamTS-B* loss-of-function mutation section), *UAS-EGFR^A887T^* (constitutively active EGFR – BL-9533), P-element *EY02397* (X chr. – BL-16538), *Df(1) Exel6236*

### Generation of *AdamTS-B* loss-of-function mutation

An imprecise excision of P-element *EY02397* (BL-16538) was used to generate the loss-of function allele of *AdamTS-B (AdamTS-B^x145^)*. Approximately 200 w^−^ males were used for further analysis. *AdamTS-B^x145^* is embryonic lethal. *AdamTS-B^x145^* was balanced over a *FM7, ftz lacZ* balancer and stained with β-Galactosidase (β-Gal) to distinguish homozygotes. To determine the extent of the deletion generated through the imprecise excision, PCR analysis was performed on 50 heterozygous flies each of WT (CS), *AdamTS-B^x145^*, and a deficiency line that removes *AdamTS-B* [*Df(1) Exel6236*] using primers along the genomic region of *AdamTS-B* (Fig. 1I and Table S1).

### Wholemount *in situ* hybridization

Wholemount *in situ* hybridization on embryos was performed as described previously (Azpiazu and Frasch, 1993; Knirr et al., 1999; Reuter and Scott, 1990). The following digoxigenin-labeled RNA probes were used: *AdamTS-B* cDNA (GH22104) and lacZ.

### Immunohistochemistry and immunofluorescence

Immunohistochemistry and immunofluorescence were performed as described previously (Azpiazu and Frasch, 1993; Knirr et al., 1999; Reuter and Scott, 1990). The following primary antibodies were used in this study: rabbit anti-b-galactosidase (1:3000, MP Biochemicals #085597-CF), rabbit anti-GFP (1:5000, Thermo Fisher A-6455), mouse anti-2A12 [1:10, Developmental Studies Hybridoma Bank (DSHB)], mouse anti-Crb (1:10, fluorescence, 1:100, HRP - DSHB Cq4), mouse anti-Tgo (1:2, DSHB), anti-Piopio (1:20, gift from Dr. Marcus Affolter, University of Basel, Switzerland), 488-WGA (1:1500, Molecular Probes), anti-DIG-AP (1:2000, Roche #11093274910). The following secondary antibodies were used in this study: biotinylated anti-rabbit (1:500), biotinylated anti-mouse (1:500), goat anti-rabbit-488 (1:500), goat anti-rabbit-488 (1:500), goat anti-mouse-488 (1:500), DAPI (1:1000).

### Microscopy

A Nikon Eclipse CiL compound light microscope using differential interference contrast optics was used at 20X magnification. Brightfield images were analyzed using Nikon Elements software. A Nikon Eclipse Ti Confocal Microscope was used to image fluorescence-stained embryos at 40X and 60X objective lenses. All fluorescent images were analyzed and processed using Nikon Elements Ar software.

### Statistical analysis

A G-test of independence was used to compare *AdamTS-B* overexpression defects compared to WT embryos.

## Supplementary Material



10.1242/biolopen.062602_sup1 Supplementary information
