# Supplementary material for: The extracellular protease AdamTS-B is a dosage-sensitive regulator of unicellular tracheal branch morphogenesis
Source: Biol Open. 2026 Aug 14;15(8):bio062602. doi: 10.1242/bio.062602 (PMC13552476; doi:10.1242/bio.062602)
Supplement: Supplementary file 1 [file biolopen-15-062602-s1.pdf]

Table S1. Primers, primer pairs, and PCR sizes used in *AdamTS-B* excision analysis

primer name	sequence 5' to 3'
F4	ggcgtggcagacagtctgcaatca
F3	ggacggatggatgtctggacaatg
F2	gttgttgctgtctttgaagcgccg
F1	tcgactgcaaatcggctgctgtgg
R1	gattgtgtgtggcatacggaggcc
R2	accacgaccagccaatagtggagc
R3	tgggtatcgtgttccgagttcagg
R4	aacttggtgtgcggaactctgctg
R5	gcactgcatccattctcccagcag
R6	gtgccgaaggacaagattgcctac
R7	gcgagggtgagtttaagcaaccag
cF	cgcgaaccgcgtcttgttcagaaa
cR	gtgctacttggtcctgcagatcgg

primer pair	PCR size (bp)
F1, R1	400
F1, R2	900
F3, R1	900
F3, R2	1400
F3, R3	1600
F3, R4	2300
F4, R4	2500
F1, cR	6200
F1, R5	6600
F4, cR	6900
F3, R5	7100
F4, R5	7300
F4, R6	7400
F4, R7	7600

Biology Open • Supplementary information
