# Supplementary material for: The extracellular protease AdamTS-B is a dosage-sensitive regulator of unicellular tracheal branch morphogenesis
Source: Biol Open. 2026 Aug 14;15(8):bio062602. doi: 10.1242/bio.062602 (PMC13552476; doi:10.1242/bio.062602)
Supplement: Supplementary file 2 [file biolopen-15-062602_review_history.pdf]

# The extracellular protease AdamTS-B is a dosage-sensitive regulator of unicellular tracheal branch morphogenesis

Abigail Thuringer, Elizabeth Steinmetz, Muna Abdullahi and Afshan Ismat
10.1242/bio.062602

**Editor**: Tristan Rodríguez

**Review timeline**

| | |
|---|---|
| Submission to sister journal: | 27 February 2026 |
| Editorial decision at sister journal: | 31 March 2026 |
| Transfer to Biology Open: | 15 July 2026 |
| Accepted: | 16 July 2026 |


---

**Original submission to sister journal**

First decision letter

MS Title: The extracellular protease AdamTS-B is a dosage-sensitive regulator of unicellular tracheal branch morphogenesis

Authors: Abigail Thuringer, Elizabeth Steinmetz, Muna Abdullahi and Afshan Ismat

I have now received all the referees' reports on the above manuscript, and have reached a decision. I am sorry to say that the outcome is not a positive one. The referees' comments are appended below.

As you will see, the referees raise some significant concerns about your paper, and are not strongly in favour of publication. Having looked at the manuscript myself, I agree with their views, and I must therefore, reject your paper.

I do realise this is disappointing news, but we receive many more papers than we can publish, and we can only accept manuscripts that receive strong support from referees.

I do hope you find the comments of the referees helpful, and that this decision will not dissuade you from considering our journal for publication of your future work. Many thanks for sending your manuscript to us.

Reviewer 1

Comments for the author

In this manuscript, Ismat and colleagues investigate the role of AdamTS-B, a matrix metalloprotease, in the branching morphogenesis of the embryonic tracheal system in Drosophila. The authors show that AdamTS-B is expressed in tracheal cells during embryogenesis and generate a putative null mutation in the gene. The mutant tracheal phenotype exhibits shortened branches and possible defects in cell intercalation. In contrast, overexpression of AdamTS-B in the trachea leads to cyst-like structures and hyper-elongation of certain branches. These findings suggest that AdamTS-B plays an important role in normal tracheal morphogenesis.
While the identification of a new mutant phenotype is of interest to researchers studying matrix proteases and tracheal development, there are several significant shortcomings in the current manuscript that make it difficult to draw firm conclusions about AdamTS-B function.

© 2026. Published by The Company of Biologists under the terms of the Creative Commons Attribution License (https://creativecommons.org/licenses/by/4.0/).

SUGGESTIONS TO AUTHORS

1. Proposed mechanism involving apical ECM remodeling
The authors propose that AdamTS-B regulates tracheal tube shape and cell intercalation by degrading the apical extracellular matrix (ECM) in the lumen. This hypothesis is based on previous work showing that ZP-domain proteins such as Pio and Dpy limit cell intercalation (Jaźwińska et al., 2003). However, this argument is not sufficiently supported. The substrate(s) of AdamTS-B are not identified in this study, and it remains unclear to what extent the phenotypes of AdamTS-B loss- or gain-of-function resemble those of pio or dpy mutants. Additional experimental evidence is required to substantiate this model.
2. Proposed involvement of EGFR signalling
The authors also suggest that AdamTS-B may function through the EGFR signaling pathway. This claim is based primarily on a reported phenotypic resemblance between AdamTS-B mutants and EGFR[A887T] overexpression. This is not convincing. A more rigorous analysis—such as epistasis experiments and the use of pathway-specific markers—is necessary to support a functional link between AdamTS-B and EGFR signaling.
3. Characterization of the AdamTS-B mutation
The newly generated AdamTS-B[x146] allele is insufficiently described. Neither Figure 2 nor the Methods section provides a molecular characterization of this mutation. Ideally, the mutation should be defined at the molecular level (e.g., a deletion affecting part or all of the AdamTS-B coding sequence while leaving neighboring genes intact). The authors should verify and clearly document the nature of this allele.
4. Localization of AdamTS-B-GFP
The authors report that AdamTS-B-GFP, expressed under the btl enhancer, localizes predominantly to the tracheal lumen and cytoplasm of tracheal cells. However, accumulation in the lumen is expected given its enclosed nature. This observation does not exclude the possibility that AdamTS-B is also secreted basally and rapidly diffuses into the body cavity. Additional experiments would be needed to clarify its secretion dynamics and localization.
5. Inconsistent use of markers and quantification
The analysis lacks consistency in both marker selection and quantification criteria. For mutant analysis, Crumbs (Crb) staining is used to visualize apical junctions and assess cell intercalation. In contrast, overexpression experiments rely on luminal marker 2A12 and membrane marker myrGFP. Moreover, different criteria are used to quantify abnormalities (e.g., Fig. 3C vs. Fig. 4G, H). This inconsistency makes it difficult to directly compare phenotypes of the loss-of-function and gain-of-function experiments that is necessary to draw cohesive conclusions about AdamTS-B function. A standardized approach to marker usage and quantification is needed.

Minor Comments
1. Figure 1
Figure 1 does not appear to provide substantial new information. It could be removed or integrated into another figure.
2. Figure 3D
Figure 3D aims to illustrate cell body movement during unicellular tube formation but is overly simplistic. It lacks information on changes in cell junctions and their relationship to the lumen. Additional detail would improve its interpretability.
3. Description of tracheal morphology
Terms such as "shortened," "bulbous," and "misshapen" tracheal branches are not clearly defined. These descriptions should be supported by quantitative measurements and high-resolution images showing tracheal cells, junctions, lumen structure, and other relevant features.

<u>Reviewer 2</u>

Comments for the author

The manuscript by Thuringer et al. investigates the role of the extracellular protease AdamTS-B during Drosophila tracheal development. The authors report that AdamTS-B is expressed in tracheal cells, generate a mutant allele, and analyze its function during tracheal formation. They propose a role for AdamTS-B in cell intercalation. Using a GFP-tagged transgene, they propose that the protein is secreted and localizes to basal protrusions. By analogy to defects observed upon

© 2026. Published by The Company of Biologists under the terms of the Creative Commons Attribution License (https://creativecommons.org/licenses/by/4.0/). 

constitutive Egfr activation, they propose that AdamTS-B acts through the Egfr pathway. They propose a model in which AdamTS-B cleaves cell-aECM connections to permit cell intercalation while simultaneously regulating Egfr activity by releasing or presenting its ligand.

Although this is an interesting hypothesis, the results presented do not support the authors' conclusions. Some of the results appear to be incorrectly interpreted or described, and none of the proposed conclusions are convincingly demonstrated by the data provided.

SUGGESTIONS TO AUTHORS

Major concerns

1) AdamTS-B loss of function phenotype.
The authors generate a mutant allele for AdamTS-B and analyze the tracheal pattern at stages 14 and 15. They claim that the LT/GB branches are more bulbous, shorter, and misshapen in mutants (60%) compared with controls (30%). They also report intercalation defects at stage 15.
However, no quantification of the "bulbous/shorter/misshapen" phenotype is provided, aside from reporting its presence or absence in control and mutant embryos. Proper measurements of these defects, ideally normalised to overall embryo size/shape, are necessary to support these claims.
To evaluate intercalation, the authors use Crb as a marker. Crb is not an optimal marker for detailed assessment of intercalation. More appropriate markers, such as ECadherin or other adherens junction components, would provide a clearer and more reliable analysis.
The data presented in Fig. 3G/H are not convincing. Loops are also visible in the control image (Fig. 3G; metameres 1 and 3), suggesting that the phenotype may have been over-interpreted. A quantitative analysis is required to substantiate intercalation defects, for example, counting the number of intercellular versus autocellular stretches in the GB.
In addition, tracheal cell intercalation is a dynamic process that is not perfectly synchronized across metameres. Therefore, it is essential to analyze old embryos, in which intercalation is complete, to properly assess defects.
According to the authors' model, intercalation defects would be expected in all tracheal branches. The dorsal branches (DBs) have traditionally served as the canonical system for analyzing tracheal intercalation, as they undergo a complete transformation from intercellular to fully autocellular tubes unlike the LT/GB, where the mixture of cell arrangements can be confounding. Can the authors analyze and quantify cell intercalation in the DBs to strengthen their claims?

2) AdamTS-B gain of function phenotype.
The authors overexpress AdamTS-B in the trachea and report several defects:
- Extralong GBs. This is unclear from the images presented. The quantifications only evaluate the penetrance of the defect, but not the defect itself. It would be necessary to measure the length of the GBs in control and AdamTS-B-overexpressing embryos in relation to embryo size.
On the other hand, I am unsure that (some of) the branches indicated with red arrowheads in Fig. 4B are actually GBs, rather than terminal branches (e.g., LH, LE…, see Manning and Krasnow, 1993).
- Luminal cysts. This phenotype seems clear in the images provided and is well quantified. However, higher resolution imaging would be needed, in combination with cell markers (apical, basal), to understand the cellular organization of these cysts.
- Ectopic branches. As the authors point out, this would indeed be a remarkable phenotype. Unfortunately, there is no quantification or clear documentation of it. What is shown in Fig. 4D could simply represent the formation of three terminal LGs, which often occurs (see (Uv et al., 2003, Trends Cell Biol, 13, 301-9.), Fig. 1).
- Extra and thicker basal protrusions. This effect is not quantified relative to the control. Moreover, its interpretation is uncertain: how does this relate to the apical secretion of the protease?

3) AdamTS-B subcellular localization
The authors investigate the subcellular localization of AdamTS-B by expressing AdamTS-B-GFP in the trachea.
On the one hand, they claim that the protein localizes to the lumen of unicellular branches. However, this is not clear from the images provided. In fact, the opposite seems more likely, as there is extensive "red-luminal" signal without corresponding "green AdamTS-B" signal. At the resolution shown, AdamTS-B appears to localise throughout the cell, likely abutting the apical

© 2026. Published by The Company of Biologists under the terms of the Creative Commons Attribution License (https://creativecommons.org/licenses/by/4.0/). 

region. True luminal localisation is usually very evident (see, for example, ANF-GFP in Tsarouhas et al., 2007, Dev Cell, 13, 214-25.).
On the other hand, it is unclear to what extent this experiment reflects the actual subcellular distribution of endogenous AdamTS-B rather than showing a pattern due to overexpression, potentially resulting in cellular overload of the protein.

4) AdamTS-B and EGFR
Based solely on phenotypic similarities, the authors claim that AdamTS-B acts through Egfr. The authors analyze Egfr overactivation and report that the GBs are elongated. However, no quantification is provided to support this claim. They also observe cysts in EgfrCA embryos. However, since no detailed cellular analysis of the cysts is presented, either in AdamTS-B overexpression or in EgfrCA conditions, it is not possible to conclude that these structures arise through identical mechanisms.
The authors propose that AdamTS-B acts through the Egfr pathway. If this is the case, does downregulation of Egfr reproduce the defects of AdamTS-B mutants? Can the expression of EgfrCA rescue the intercalation defects indicated in AdamTS-B mutants?

5) Model
The model proposes that "AdamTS-B functions within the aECM of tracheal tubes, where it would normally sever connections between tracheal cells and the aECM". However, no evidence is provided to support this, and the aECM is not analyzed anywhere in the report.
The model also hypothesises that "AdamTS-B functions through the Egfr pathway in the lumen to regulate this cell intercalation process, either by physically freeing up the Egfr to increase Egf ligand binding, or by physically helping one or more of the Egf ligands bind to Egfr." Again, there is no evidence supporting this mechanism, and Egfr ligands are not analyzed in this work. What evidence supports the idea that the Egfr ligand/s are modulated by AdamTS-B? Interestingly, it has been shown that different RTKs can induce cyst formation in the trachea (Jeon et al., 2012, Biol Open, 1, 548-58). Do the authors propose that the protease regulates the ligands of all of them?

Minor issue

1) Tracheal placodes are specified at stage 10. The expression of AdamTS-B shown may therefore correspond to stage 10, rather than 9. The pattern shown in Fig 1 at stage 12 and 14 is puzzling, as the gene does not seem to be expressed in all tracheal cells (dorsal part of the tracheal system, including dorsal branches and dorsal trunk, is not detected). Is this the case?

---

**Transfer to Biology Open**

Author response to reviewers' comments

**EDITOR'S COMMENTS**

I believe that the vast majority of the reviewers' comments can be addressed by modifying the claims presented in the manuscript. These should be adjusted so that they are more circumspect, and more accurately reflect the results that are presented in the manuscript. I do not think that there is a requirement for additional experimentation. However, should the requested experimental results be available such that the original claims can be adequately supported, then please provide these data in the revised manuscript.

Author response: We agree with this assessment of our manuscript. We have completed a more careful analysis of our LOF and overexpression mutants which included some more experiments at late stages. Specifically, we have found no evidence of cell intercalation defects in the *AdamTS-B* LOF mutants using stage 16 embryos and examining both dorsal branches (DBs) and ganglionic branches (GBs) using *btl*-GAL::*UAS-myrGFP* (to mark tracheal cell membranes) and Tgo (to mark tracheal nuclei). Additionally, we could not conclude any genetic or physical interaction between AdamTS-B and the EGFR pathway and so have revised our manuscript to state there may be a possible interaction. Overall, we have adjusted our manuscript to be more circumspect and reflect the data presented.

© 2026. Published by The Company of Biologists under the terms of the Creative Commons Attribution License (https://creativecommons.org/licenses/by/4.0/).

Please see our responses to all the major and minor concerns from Reviewers 1 and 2 below.
SUGGESTIONS TO AUTHORS – Reviewer 1
Major concerns

1. Proposed mechanism involving apical ECM remodeling
The authors propose that AdamTS-B regulates tracheal tube shape and cell intercalation by degrading the apical extracellular matrix (ECM) in the lumen. This hypothesis is based on previous work showing that ZP-domain proteins such as Pio and Dpy limit cell intercalation (Jaźwińska et al.,2003). However, this argument is not sufficiently supported. The substrate(s) of AdamTS-B are not identified in this study, and it remains unclear to what extent the phenotypes of AdamTS-B loss- or gain-of-function resemble those of pio or dpy mutants. Additional experimental evidence is required to substantiate this model.

Author response: We agree with this reviewer's critique. We did a more careful analysis looking at stage 16 embryos when cell intercalation is complete (using *btl*- GAL::*UAS-myrGFP* to mark tracheal cell membranes and Tgo to mark tracheal nuclei) and did not find any defects in cell intercalation in the dorsal branches (DBs) or any defects in the GBs (see revised Figure 2). We also included Crb staining at stage 14 and, after a more careful analysis, we do not see any obvious defects in the DBs at stage 14, but do see somewhat shorter GBs. This slight defect seems to be transient as the GBs are normal at stage 16. Therefore, we have removed our quantification and have altered the manuscript to reflect our more careful analysis.

2. Proposed involvement of EGFR signaling
The authors also suggest that AdamTS-B may function through the EGFR signaling pathway. This claim is based primarily on a reported phenotypic resemblance between AdamTS-B mutants and EGFR[A887T] overexpression. This is not convincing. A more rigorous analysis—such as epistasis experiments and the use of pathway-specific markers—is necessary to support a functional link between AdamTS-B and EGFR signaling.

Author response: Since there is just a very slight defect in the *AdamTS-B* LOF (see revised Figure 2), our experiments were inconclusive of AdamTS-B functioning through the EGFR pathway. First, we expressed secreted Spi (UAS-sSpi) in the trachea in the *AdamTS-B[x145]* mutant background, but did not see any change in defect using the Crb tracheal marker (data not shown). We only found somewhat shorter GBs in *AdamTS-B[x145]* and *btl*-GAL4::*UAS-AdamTS-B[RNAi]* mutants using Crb (see revised Figure 2). We also expressed secreted Spi (sSpi) and uncleaved Spi (mSpi) in the trachea and did not see any obvious defects using 2A12. We attempted to express either EGFR[A887T] or mSpi in the *AdamTS-B[x145]* mutant background, but these flies were very weak and did not survive long enough to collect embryos. More time would be required to do these experiments properly and systematically.

Therefore, we have changed the manuscript to suggest that there is a possible link between AdamTS-B and EGFR signaling in the trachea, but more analysis needs to be done. Butchar et al. (2012) showed a link between AdamTS-B and EGFR signaling in wing vein formation, so it is possible that this link is present in the trachea as well.

3. Characterization of the AdamTS-B mutation
The newly generated AdamTS-B[x145] allele is insufficiently described. Neither Figure 2 nor the Methods section provides a molecular characterization of this mutation. Ideally, the mutation should be defined at the molecular level (e.g., a deletion affecting part or all of the AdamTS-B coding sequence while leaving neighboring genes intact). The authors should verify and clearly document the nature of this allele.

Author response: We have completed PCR analysis on *AdamTS-B[x145]* heterozygotes using several primers (see Materials and Methods, revised Figure 1 for primer locations, and Supplemental Table 1) and have no defined deletion.

4. Localization of AdamTS-B-GFP
The authors report that AdamTS-B-GFP, expressed under the btl enhancer, localizes predominantly to the tracheal lumen and cytoplasm of tracheal cells. However, accumulation in

© 2026. Published by The Company of Biologists under the terms of the Creative Commons Attribution License (https://creativecommons.org/licenses/by/4.0/).

the lumen is expected given its enclosed nature. This observation does not exclude the possibility that AdamTS-B is also secreted basally and rapidly diffuses into the body cavity. Additional experiments would be needed to clarify its secretion dynamics and localization.

Author response: After more careful analysis, we also conclude that AdamTS-B-GFP seems to localize intracellularly and at basal protrusions in tracheal cells and not very much (if at all) in the lumen. We also show that the branch cysts present in the *AdamTS-B* overexpression trachea are not luminal (do not express two luminal markers Pio and WGA) but do seem to express higher levels of AdamTS-B-GFP (see revised Figures 3 and 4).

5. Inconsistent use of markers and quantification
The analysis lacks consistency in both marker selection and quantification criteria. For mutant analysis, Crumbs (Crb) staining is used to visualize apical junctions and assess cell intercalation. In contrast, overexpression experiments rely on luminal marker 2A12 and membrane marker myrGFP. Moreover, different criteria are used to quantify abnormalities (e.g., Fig. 3C vs. Fig. 4G, H). This inconsistency makes it difficult to directly compare phenotypes of the loss-of-function and gain-of-function experiments that is necessary to draw cohesive conclusions about AdamTS-B function. A standardized approach to marker usage and quantification is needed.

Author response: We agree with this critique. We performed a more careful analysis using stage 16 embryos when cell intercalation was complete (using *btl*-GAL::*UAS- myrGFP* to mark tracheal cell membranes and Tgo to mark tracheal nuclei). We did not find any cell intercalation defects in the *AdamTS-B* LOF mutants (see revised Figure 2). We also used the same analysis on *AdamTS-B* overexpression embryos and did not find any cell intercalation defects (see revised Figure 3). We used Crb to look at earlier trachea in the *AdamTS-B* LOF (see revised Figure 2) and overexpression (data not shown since there was no difference between *AdamTS-B* overexpression and WT). We used 2A12 for LOF (data not shown since there was no difference between LOF and WT) and overexpression (see revised Figure 3).

**Minor Comments**
1. Figure 1 does not appear to provide substantial new information. It could be removed or integrated into another figure.

Author response: We have combined Figure 1 and Figure 2 into the revised Figure 1.

2. Figure 3D aims to illustrate cell body movement during unicellular tube formation but is overly simplistic. It lacks information on changes in cell junctions and their relationship to the lumen. Additional detail would improve its interpretability.

Author response: We have adjusted our interpretation (see response to Major comment 1). After a more careful analysis, we found only a slight defect in the GB at stage 14 using Crb. Therefore, any interpretation we had about cell intercalation was too strong and we have removed Figure 3D from the revised manuscript to reflect this new interpretation of our data.

3. Description of tracheal morphology: Terms such as "shortened," "bulbous," and "misshapen" tracheal branches are not clearly defined. These descriptions should be supported by quantitative measurements and high-resolution images showing tracheal cells, junctions, lumen structure, and other relevant features.

Author response: Please see response to major comment 1 and response to minor comment 2 above. We have removed the quantification from our analysis.

**SUGGESTIONS TO AUTHORS - Reviewer 2**

**Major concerns**
1) AdamTS-B loss of function phenotype.
The authors generate a mutant allele for AdamTS-B and analyze the tracheal pattern at stages 14and 15. They claim that the LT/GB branches are more bulbous, shorter, and misshapen in mutants (60%) compared with controls (30%). They also report intercalation defects at stage 15. However, no quantification of the "bulbous/shorter/misshapen" phenotype is provided, aside from

© 2026. Published by The Company of Biologists under the terms of the Creative Commons Attribution License (https://creativecommons.org/licenses/by/4.0/).

reporting its presence or absence in control and mutant embryos. Proper measurements of these defects, ideally normalised to overall embryo size/shape, are necessary to support these claims. To evaluate intercalation, the authors use Crb as a marker. Crb is not an optimal marker for detailed assessment of intercalation. More appropriate markers, such as ECadherin or other adherens junction components, would provide a clearer and more reliable analysis. The data presented in Fig. 3G/H are not convincing. Loops are also visible in the control image (Fig.3G; metameres 1 and 3), suggesting that the phenotype may have been over-interpreted. A quantitative analysis is required to substantiate intercalation defects, for example, counting the number of intercellular versus autocellular stretches in the GB. In addition, tracheal cell intercalation is a dynamic process that is not perfectly synchronized across metameres. Therefore, it is essential to analyze old embryos, in which intercalation is complete, to properly assess defects. According to the authors' model, intercalation defects would be expected in all tracheal branches. The dorsal branches (DBs) have traditionally served as the canonical system for analyzing tracheal intercalation, as they undergo a complete transformation from intercellular to fully autocellular tubes unlike the LT/GB, where the mixture of cell arrangements can be confounding. Can the authors analyze and quantify cell intercalation in the DBs to strengthen their claims?

Author response: We agree with this reviewer's critique. We did a more careful analysis looking at stage 16 embryos when cell intercalation is complete (using *btl-* GAL::*UAS-myrGFP* to mark tracheal cell membranes and Tgo to mark tracheal nuclei) and did not find any defects in cell intercalation in the dorsal branches (DBs) or any defects in the GBs (see revised Figure 2). We also included Crb staining at stage 14 and, after a more careful analysis, we do only see slight defects in the GBs at stage 14. Therefore, we have removed our quantification and have altered the manuscript to reflect our more careful analysis.

*This is the same response to reviewer #1, major comment 1.

2) AdamTS-B gain of function phenotype.
The authors overexpress AdamTS-B in the trachea and report several defects:- Extralong GBs. This is unclear from the images presented. The quantifications only evaluate the penetrance of the defect, but not the defect itself. It would be necessary to measure the length of the GBs in control and AdamTS-B-overexpressing embryos in relation to embryo size. On the other hand, I am unsure that (some of) the branches indicated with red arrowheads in Fig.4B are actually GBs, rather than terminal branches (e.g., LH, LE…, see Manning and Krasnow,1993).- Luminal cysts. This phenotype seems clear in the images provided and is well quantified.

However, higher resolution imaging would be needed, in combination with cell markers (apical, basal), to understand the cellular organization of these cysts.- Ectopic branches. As the authors point out, this would indeed be a remarkable phenotype. Unfortunately, there is no quantification or clear documentation of it. What is shown in Fig. 4D could simply represent the formation of three terminal LGs, which often occurs (see (Uv et al.,2003, Trends Cell Biol, 13, 301-9.), Fig. 1).- Extra and thicker basal protrusions. This effect is not quantified relative to the control. Moreover, its interpretation is uncertain: how does this relate to the apical secretion of the protease?

Author response: We agree with all these critiques.
Extra-long GBs: After a more careful analysis of our AdamTS-B overexpression embryos, we did not see extra-long GBs and have removed that data from our revised manuscript (see revised Figure 3).

Luminal cysts: We used Pio and WGA in our *AdamTS-B* overexpression embryos to see if the luminal cysts are indeed luminal. Both Pio and WGA do not localize to the cysts (see revised Figures 3 and 4). In addition, with a more careful analysis of our AdamTS-B localization studies using a GFP-tagged AdamTS-B (*UAS-AdamTS- B-GFP*) expressed in the trachea localize intracellularly and in basal protrusions (and not luminal). There is also a higher expression of AdamTS-B-GFP where the cyst-like structures are located (see revised Figure 4).

Extra branches: After examining a large number of embryos, we agree that the extra branches we see are not a significant defect and, therefore, we have removed that data from our revised

© 2026. Published by The Company of Biologists under the terms of the Creative Commons Attribution License (https://creativecommons.org/licenses/by/4.0/).

manuscript.

Extra and thicker basal protrusions: After a more careful analysis, we have concluded that the thicker protrusions we originally saw may have been an artifact of the level of exposure during imaging. Therefore, we have removed this data from our revised manuscript.

3) AdamTS-B subcellular localization.
The authors investigate the subcellular localization of AdamTS-B by expressing AdamTS-B-GFP in the trachea. On the one hand, they claim that the protein localizes to the lumen of unicellular branches. However, this is not clear from the images provided. In fact, the opposite seems more likely, as there is extensive "red- luminal" signal without corresponding "green AdamTS-B" signal. At the resolution shown, AdamTS-B appears to localize throughout the cell, likely abutting the apical region. True luminal localization is usually very evident (see, for example, ANF-GFP in Tsarouhas etal., 2007, Dev Cell, 13, 214-25.).On the other hand, it is unclear to what extent this experiment reflects the actual subcellular distribution of endogenous AdamTS-B rather than showing a pattern due to overexpression, potentially resulting in cellular overload of the protein.

Author response: After more careful analysis, we also conclude that AdamTS-B-GFP seems to localize intracellularly and in basal protrusions in tracheal cells and not very much (if at all) in the lumen. We also show that the cyst-like structures present in the *AdamTS-B* overexpression trachea are not luminal (do not express two luminal markers Pio and WGA) but do seem to express higher levels of AdamTS-B-GFP (see revised Figures 3 and 4).

*This is the same response to reviewer #1, major comment 4

4) AdamTS-B and EGFR Based solely on phenotypic similarities, the authors claim that AdamTS-B acts through Egfr. The authors analyze Egfr overactivation and report that the GBs are elongated. However, no quantification is provided to support this claim. They also observe cysts in EgfrCA embryos. However, since no detailed cellular analysis of the cysts is presented, either in AdamTS-B overexpression or in EgfrCA conditions, it is not possible to conclude that these structures arise through identical mechanisms. The authors propose that AdamTS- B acts through the Egfr pathway. If this is the case, does downregulation of Egfr reproduce the defects of AdamTS-B mutants? Can the expression of EgfrCA rescue the intercalation defects indicated in AdamTS-B mutants?

Author response: We have revised the manuscript to suggest that there might also be a link between AdamTS-B and EGFR signaling in the trachea due to the similar cyst-like structures in unicellular branches, but more analysis needs to be done.
Butchar et al. (2012) showed a link between AdamTS-B and EGFR signaling in wing vein formation, so it is possible that this link is present in the trachea as well.

5) The model proposes that "AdamTS-B functions within the aECM of tracheal tubes, where it would normally sever connections between tracheal cells and the aECM". However, no evidence is provided to support this, and the aECM is not analyzed anywhere in the report. The model also hypothesizes that "AdamTS-B functions through the Egfr pathway in the lumen to regulate this cell intercalation process, either by physically freeing up the Egfr to increase Egf ligand binding, or by physically helping one or more of the Egf ligands bind to Egfr." Again, there is no evidence supporting this mechanism, and Egfr ligands are not analyzed in this work. What evidence supports the idea that the Egfr ligand/s are modulated by AdamTS-B? Interestingly, it has been shown that different RTKs can induce cyst formation in the trachea (Jeon et al., 2012, BiolOpen, 1, 548-58). Do the authors propose that the protease regulates the ligands of all of them?

Author response: We have removed that model from our revised manuscript because our experiments were inconclusive of AdamTS-B functioning through the EGFR pathway. First, we expressed secreted Spi (UAS-sSpi) in the trachea in the *AdamTS-B[x145]* mutant background but did not see any change in defect using the Crb tracheal marker (data not shown). We did not find any obvious defect in *AdamTS-B[x145]* mutants alone using Crb (see revised Figure 2). We also expressed secreted Spi (sSpi) and uncleaved Spi (mSpi) in the trachea and did not see any obvious defects using 2A12. We attempted to express either EGFR[A887T] or mSpi in the *AdamTS-B[x145]*

© 2026. Published by The Company of Biologists under the terms of the Creative Commons Attribution License (https://creativecommons.org/licenses/by/4.0/).

mutant background, but these flies were very weak and did not survive long enough to collect embryos. More time and would be required to do these experiments properly and systematically.

*This is the same response to reviewer #1, major comment 2

**Minor comment**
1) Tracheal placodes are specified at stage 10. The expression of AdamTS-B shown may therefore correspond to stage 10, rather than 9. The pattern shown in Fig 1 at stage 12 and 14 is puzzling, as the gene does not seem to be expressed in all tracheal cells (dorsal part of the tracheal system, including dorsal branches and dorsal trunk, is not detected). Is this the case?

Author response: We agree that the embryo we labeled stage 9 is actually stage 10 and have changed that in our revised manuscript (see revised Figure 1). We agree that it would be helpful to know exactly which tracheal branches express *AdamTS-B* mRNA. We tried to perform a fluorescent in situ of *AdamTS-B* mRNA with Crb, but the *AdamTS-B* mRNA expression was extremely hard to visualize due to too much background staining. Therefore, we cannot answer this question with the data we presently have.

---

First decision letter

MS ID#: bio.062602

MS Title: The extracellular protease AdamTS-B is a dosage-sensitive regulator of unicellular tracheal branch morphogenesis

Authors: Abigail Thuringer, Elizabeth Steinmetz, Muna Abdullahi and Afshan Ismat

I am happy to tell you that your manuscript has been accepted for publication in Biology Open, pending our standard publication integrity checks. It was accepted on 16th July 2026.

© 2026. Published by The Company of Biologists under the terms of the Creative Commons Attribution License (https://creativecommons.org/licenses/by/4.0/).
